# Palliative Curriculum Re-imagined: A Critical Evaluation of the UK Palliative Medicine Syllabus

**DOI:** 10.1177/1178224218780375

**Published:** 2018-05-29

**Authors:** Julian Abel, Allan Kellehear

**Affiliations:** 1 Palliative Care, Faculty of Health Studies, DHEZ Academic, University of Bradford, Bradford, UK

**Keywords:** Palliative medicine, syllabus, curriculum, training, public health, United Kingdom

## Abstract

The UK Palliative Medicine Syllabus is critically evaluated to assess its relationship and relevance to contemporary palliative care policy and direction. Three criteria are employed for this review: (1) relevance to non-cancer dying, ageing, caregivers, and bereaved populations; (2) uptake and adoption of well-being models of public health alongside traditional illness and disease models of clinical understanding; and (3) uptake and integration of public health insights and methodologies for social support. We conclude that the current syllabus falls dramatically short on all 3 criteria. Suggestions are made for future consultation and revision.

## Introduction

Palliative care is that branch of health care devoted to the care of dying people and their families. As the World Health Organization (WHO) guidance argues, it is not solely about prognosis but also needs, not solely about the dying person but also their caring networks (http://www.who.int/cancer/palliative/definition/en/). Although treatment and care of dying people living with progressive and non-curable forms of life-limiting illness is a central focus, this care is not limited to physical care but also includes psychological, social, and spiritual forms of support. The reason for this broad remit is based on the widely acknowledged truism that dying is only partly a physical encounter with mortality, with most of the human drama surrounding death, dying, caregiving, and grief lying in the domains of the psychological, social, and spiritual worlds of individuals and their intimate social networks.

For these above reasons too, the speciality of palliative medicine is commonly and internationally seen as a *team* specialism – a profession usually working closely with other professions in the health services – nurses, family medical (general practitioner [GP]) colleagues, allied health workers, volunteers, social workers, counsellors, to name only a few of these collaborators. For those working in palliative care, levels of competency are aimed at ensuring each professional can perform a wide variety of roles where more severe problems may need specialist support. For example, family doctors may need specialist support for symptom science from palliative care specialists: social matters might be addressed through actions by social work colleagues and volunteers. Psychological challenges might be addressed through relevant referrals to colleagues in psychology or psychiatry. Spiritual challenges might be referred to a chaplain or pastoral care colleague. In general, non-medical challenges have frequently been referred to as ‘psychosocial issues’ – a phrase and a practice that commonly sees greater emphasis on personal challenges for individual patients rather than social challenges for and inside their networks and supports.

Furthermore, this emphasis on the team approach results, almost by definition, in a focus on professional interventions alone (ie, direct services) whatever the profession, and when these are deployed, commonly as a short, if regular, face-to-face style encounter (ie, mostly an acute care intervention). In the last 10 years of palliative care development, this solely professional services approach to human problems has come under increasing scrutiny, criticism, and revision.

There is now wide recognition, in recent and current policy documents described below, that dying people, caregivers, and the bereaved have very specific public health needs – depression, social isolation, lost work or school days, or disturbed social relationships. Most of these troubles are explicitly social in nature and are amenable to prevention, harm reduction, and early intervention strategies. Broadly speaking, symptom control issues and addressing psychological, social, and spiritual needs through professional service interventions falls into the category of harm reduction. However, ameliorating symptoms or addressing need is quite a different matter from health promotion and well-being.^1^ Active participation in enhancing well-being has been at the forefront of recent developments in the new public health approach^2^ to palliative care. Supportive networks surrounding patient and carer form a rich source of meaning, actions, and value to all involved, which persists years into bereavement.^3^ Palliative care services have an important role in stimulating these communities of support, whether they are individual networks surrounding the patient, in the workplace, educational institutions, or neighbourhoods.

Furthermore, there is now a widely acknowledged challenge that palliative care has significant access issues.^4^ Palliative care has long been identified with cancer care but now needs to address a rapidly ageing population characterized by chronic illness and multi-morbidity. Frailty, organ failure, and neurological conditions often eclipse, or exist alongside malignant conditions for many of today’s dying. Compounding these medical issues of access are further social challenges of lengthy dying trajectories, regional access from rural and remote areas, and addressing marginal populations with special palliative needs, for example, prison populations, homelessness, or ethnic/religious minorities with complex health and bereavement needs.

In the last 10 years, our epidemiological, sociological, and health services understandings have changed dramatically. Is the training that palliative care doctors receive keeping up with these changes? To what extent can we be confident that present and future palliative care consultants are prepared to meet these new public health challenges for the exponentially rising groups of ageing, dying, caregiving, and bereaved populations?

The aim of this article is to critically evaluate the key syllabus used in the UK training of palliative care doctors. We assess this document against current practice guidelines widely available in palliative care and public health policy sectors. We argue that the current syllabus reflects an over-attention to clinical concerns of harm reduction and lacks acknowledgement – or simply under-emphasizes – crucial public health and social concern. There is a particular lack of emphasis on promotion of health and well-being. Health promotion in the context of palliative care does not refer to merely altering the disease trajectory. Rather, it refers to a wide variety of ways of creating and enhancing positive meaning, action, and value in the experiences of death, dying, loss, and caregiving for all those involved. In these ways, the current UK syllabus inadequately prepares its trainees for the main social and public health challenges of living while dying, or living with long-term caregiving, grief, and bereavement.

In support of these observations, we organize the article in the following way. First, we will provide a brief overview of current, and publicly acknowledged, social and public health challenges identified in recent palliative care policy literature. Second, we summarize the UK Palliative Medicine Syllabus. We then proceed to outline the basic problems with the syllabus as a whole, taking particular note of its public health omissions and deficits. We conclude with some final reflections and suggestions for this curriculum’s future development.

## Current Policy Context for UK Palliative Medicine

Recent palliative care policy in the United Kingdom has consistently emphasized and highlighted the need for attention to social aspects of care. As early as the 2008 End of Life Care Strategy^5^ – a policy that preceded the Palliative Medicine Curriculum we are about to assess – there is a clear stipulation that all palliative care from all professions must support carers and the bereaved. Palliative care is not simply care of those living with a life-limiting illness but it also includes care for those caring for the seriously ill, acknowledging that such care could last years in cases of modern metastatic cancer or dementia. Caregivers often experienced morbidities associated with their lifestyle similar to the co-morbidities of those they care for. Furthermore, the bereaved often live with major morbidities and risk to life as do long-term carers – depression and job loss were commonly noted as incidences of suicide and sudden death for the bereaved and caregivers.^6-8^ Social supports are an essential part of palliative care provision and an explicit requirement of all their related services such as bereavement, cancer, and aged care.

The 2011 NICE Guidelines^9^ were similarly adamant in their Quality Statements 5 to 7 that stipulated: people at the end of life must feel able to maintain social participation *according to their preferences* and to feel emotionally supported. The NHS Action for End of Life Care for England (2014-2016)^10^ describes what it calls ‘The House of Care’ – a care metaphor that discusses the essentials of palliative care arguing for the centrality of support planning, partnership working, and engagement and involvement with compassionate communities – a public health phrase for community development and partnership.^11^

In 2015, a further iteration of the 2008 End of Life Care Strategy emerged as the ‘Ambitions for Palliative and End of Life Care: A National Framework for Action’ policy document.^12^ In this policy – which included a consultation with the Royal College of Physicians – 6 ‘ambitions’ were described and the sixth was the exhortation that ‘Each community is prepared to help’. Everyone has a role to play in end-of-life care – not simply or solely health professionals.

It is looking to these national policies and guidance documents that we gather up a criteria to assess the adequacy and relevance of the UK Palliative Medicine Syllabus. What does this syllabus look like and how well does it mirror and promote the policy values and assumptions of these recent documents? Has palliative medical training re-oriented to the ‘new’ public health challenges in these policies? Does the syllabus reflect that fine balance – required nationally for the last 10 years – of clinical and community practice, of the requirement to be inclusive of caregivers and the bereaved, and of balancing an illness and disease model of dying and grief with an overlapping model of health and well-being?

## The UK Palliative Medicine Syllabus: Overview

The current UK Palliative Medicine Syllabus was prepared in 2010, with amendments made in 2014 and some minor administrative changes made in 2015 – a development process that parallels the timelines of the above national policy documents but departs substantially from them in content. It is a 106-page document authored by the Joint Royal College of Physicians Training Board^13^ (see https://www.gmc-uk.org/-/media/documents/2010-palliative-medicine-curriculum–amendments-2014–141015_pdf-63150821.pdf). After some preliminary pages discussing rationale, purpose, development methodology, description of the training pathway, and some basic remarks about the principle underlying the content of learning, the rest of the document outlines the curriculum for training.

The number of pages the syllabus devotes to the different topics of training is itself quite revealing for this is a good indicator of both topic priority and importance (or conversely their lack). The Introduction to ‘palliative care’ as a concept and philosophy requires 4 pages; 20 pages are devoted to aspects of physical care; 5 pages are devoted to communication matters; 4 pages are devoted to ‘psychosocial’ care; 2 pages are devoted to culture, language, religion, and spirituality; 12 pages are devoted to management and clinical governance (including quality and safety issues, audits of clinical practice, and so on); and 2 to 4 pages each are devoted to an assortment of other topics such as attitudes and responses to other doctors and professionals, research, self-learning, teamwork, legal frameworks, and ethics.

The syllabus itself is broken into 3 major sections headed by an aim or several aims to be addressed by the section which is then divided into essential learning outcomes – ‘Knowledge’, ‘Skills’, and ‘Behaviours’. The formal part of the syllabus is then followed by a discussion of Learning and Teaching topics – description of the training programme, supervised work experience, teaching and learning methods, discussions of pedagogy, and research options within the programme. This section on learning and teaching is followed by discussions about assessment and timetabling. This section is followed by others on supervision and curriculum implementation, review, and updating. The final section devotes itself to a brief discussion of compliance with equality and diversity legislation (The Equality Act 2010). Appendices provide a list of contributors to the syllabus and a graphic layout for feedback mechanisms for students taking the training. Most of the comments and observations we make in this article are confined to the syllabus section of this document.

## General Problems

Public health is not simply identifying the social determinants of health and identifying social and clinical epidemiological trends. The surveillance functions of public health tend to stereotype public health by identifying the field solely with these functions. ‘Health services research’ further complicate our academic understanding of ‘public health’ by over-identifying this sub-field with intervention/implementation studies and their evaluation methodologies. However, public health concerns are also about *community practices* – about public education, community development and engagement, social ecology (creating healthy environments), development of personal skills, policy development, and social marketing (‘selling’ health, safety, or well-being to communities). Away from university research centres, much public health work consists of the promotion of these different community practices that support the health of ‘publics’. Together all these strands of public health activity bear upon a handful of health concerns – addressing patterns of morbidity and mortality, and alongside these, promoting patterns of health and well-being. For problems of morbidity and mortality, most public health practice is aimed at prevention, harm reduction, and early intervention. Overlapping with these problem-based concerns is health promotion – the need to promote health and well-being as the optimum strategy against illness, disability, and accident.

For some time in palliative care, health promotion was viewed as an oxymoron – a contradiction in terms.^14^ This understanding has witnessed dramatic change with a strong evidence base within the last 20 years of palliative care literature.^15-20^ The field of palliative care now acknowledges that there are obvious co-morbidities associated with dying, caregiving, and bereavement especially as these pertain to the social (not simply clinical) epidemiology of these experiences – depression, anxiety, loneliness, missed work days, social rejection, and job loss among many other social problems. Most of these are not technically ‘psychosocial’ problems – they are social problems relating to poor public and personal support networks, poor or absent school or workplace policies, poor or absent partnerships between health services, social care providers, and our communities. Unaddressed, these problems result in poor outcomes in a number of different domains, which may last for years and have an associated mortality. Active participation in reducing harm and promoting well-being can have a dramatic impact on lifelong problems.

Compounding this growing awareness about social ‘problems’ is a lack of acknowledgement concerning the parallel health and well-being aspects of dying, caregiving, and bereavement – the commonly observed growth of love, intimacy, and friendship; the will to meaning and well-being found in relationships during dying, caregiving, or grief; or the powerful support role of community during this time. Palliative care services can actively participate in supporting interventions in these areas, including helping to develop compassionate communities and supporting civic programmes.^21^ How well does the current syllabus reflect these contemporary public health insights?

There are 13 initial critical observations to be made of the current syllabus. These observations reflect *general deficits* in the overall document. They are highlighted particularly because they ignore the personal and community dimensions of dying, caregiving, and bereavement and are remarkable for that fact:

1. In a 106-page document only 4 pages are devoted to psychosocial care and 1.5 pages to public health concerns, and this despite the claim that palliative medicine must ‘optimize’ (among other things) social support (p. 3).
2. Consultation for the syllabus was designed in close consultation with doctors ‘from closely aligned specialities such as pain medicine and oncology’ (p. 3) but clearly not from public health or geriatric medicine.
3. The claim about the importance of ‘shared care’ does not include sharing that care with community or other civic organisations and sectors that work with or alongside the dying, caregivers, or the bereaved.
4. The concept of community is commonplace in the document but its meaning as a social influence on health and well-being appears trivial and isolated (p. 28), eclipsed as it is by larger concerns with other less public health orientated meanings. The syllabus is more concerned with community as a potential work site (pp. 3, 82, 83, 85); as a place where a service might be located (pages 14, 32) or where patients might be found (p. 14); as a site for risk management of communicable diseases (p. 30); or as a site for managing one’s own risk and personal safety when visiting (p. 48); and finally, as a site where one might assess ‘health needs’ or ‘community action and advocacy’ without specifying what these might be (pp. 70, 72).
5. Fear and fear of death – the subject of endless academic and fictional treatments in literature, poetry, theatre, and film – receives one mention (p. 26). It rates as a problem on par with anxiety and insomnia.
6. Love, intimacy, and friendship are given no mention – arguably the most important factors in the promotion of health and well-being at the end of life, and for bereaved people, and important protectors against, if not breaks upon, risk of poor psyche and spirit.
7. Conflict, on the other hand, between patients and professionals and between professionals themselves, is mentioned 20 times.
8. Well-being is mentioned only twice and one of these refer to self-care management for professionals.
9. Hope is mentioned twice in the whole document (p. 50) but happiness or its opposite, despair, do not appear as concerns.
10. Meaning – a crucial existential and religious exercise for most people at the end of life and in bereavement (as it often is during the course of life itself) – is mentioned only once (p. 49) and is linked only to illness not to death, nor to loss, nor to life or human bonds.
11. Although there are 2 pages devoted to the important topics of religion and spirituality, rather inexplicably there is no mention of god – a subject at the very centre of the majority of world religions and its many living and dying adherents.
12. There is mention of confusional states and hallucinations but no mention of common and well-documented experiences near-death that do not conform to these psychiatric categories – near-death experiences, deathbed visions, or visions of the bereaved. The prevalence of these experiences at the end of life is significant and varies from 10% to 80%.^22^ Their omission is not explained.
13. As a medical training document, the co-morbidities of dying, caregiving, or bereavement enjoy absolutely no profile in the syllabus. There is correspondingly a clear absence of any public health practice methods to address these. There is scarcely a mention of the health and well-being aspects and implications for these populations.

This final observation brings us to the specifically public health deficits to note in the syllabus.

## Public Health Deficits

Section 2.15 is devoted to ‘public health related to palliative care’. This is a page and a half of text broken into further sections on the required knowledge, skills, and behaviours expected from this specific field. The remarkable facts to note about this section are as follows:

1. The knowledge requirements are not specifically related to life-limiting illness, ageing, caregiving, or bereavement. Trainees are expected merely to understand the public health factors that influence general health and illness.
2. There is no requirement to understand the co-morbidities or mortalities – the social and clinical epidemiology – of living with advanced ageing, dying, long-term caregiving, or grief and bereavement.
3. There is no requirement to understand key concepts (prevention, harm reduction, early intervention) or their methods and to link these to palliative care (community development, social ecology, death literacy, social marketing, health promotion).
4. As an extension of the general problem with the syllabus, the idea of community is left vague and unpacked. There is no mention of the role of palliative medical leadership in relation to school or workplace policies or advice; no mention of the potential influence of palliative medicine on church or temple activities for support in palliative, bereavement, or aged care; no mention of any outreach role for prisons or the homeless despite explicit recommendation to understand that role elsewhere in the syllabus (p. 42).
5. Although trainees are expected to have an understanding of ‘the effects of addictive and self-harming behaviours on personal health’, they are apparently not required to have an equally important understanding of the effects of social supports on personal health and well-being.
6. Although trainees are required to understand the ‘principles of mapping service provisions and gaps’, this does not extend to mapping civic and neighbourhood social supports.
7. Although trainees are required to understand the influence of culture and beliefs on health perceptions, they are not required to apply this to death, dying, caregiving, or grief and loss.
8. Among the modest number of skills trainees are required to accrue by studying this part of the syllabus one of them is to ‘identify opportunities to improve access to palliative care’ – and not it should be noted – to identify opportunities to improve wider social supports where patients and their families live and work.
9. Trainees are to ‘work collaboratively with professional colleagues’ but not apparently with community leaders, and yet it is these leaders who are crucial in leveraging and maintaining the major civic sources of support for those living with life-limiting illness, caregiving, and bereavement.
10. Trainees are expected to ‘counsel patients’ (but not families) about their ‘ideas, concerns and social networks’ but if they do not have any, or their networks are minor, trainees are not urged to consult anyone else.
11. As a doctor, one should recognize a responsibility for ‘promoting a healthy approach to life and work’ but not apparently while living with a life-limiting illness, during long-term caregiving, or through the process of grief and bereavement.
12. Ironically for public health advocacy, trainees are expected to engage in ‘effective team-working’ but not support building, and to engage in ‘service development’ but not community development.
13. The public health section of the palliative medicine syllabus demonstrates poor familiarity with crucial concepts and methods in general public health policy and practice. Worse still, the syllabus evidences no familiarity with the established health-promoting or public health literature devoted specifically to palliative care policy and practice.

## Psychosocial Care

The section devoted to ‘psychosocial’ might be expected to say more or to further elaborate on matters to do with the ‘social’ side of palliative care – such as social support, community engagement or participation, neighbourhood relationships, or civic roles in palliative care. But it does not do so. Instead the problem of ignoring or minimizing social supports in a document that extols the need to ‘optimize’ social supports continues to fail to do so. As with the history of this use of the phrase in palliative care, ‘psychosocial’ seems to be a term that consistently fails to provide specific details about social life and instead often quickly slides all too readily into social psychology and psychotherapeutics. The supportive network in which the patient and carer live has great potential to enhance the most meaningful components at a critical time in their lives. Actively engaging, supporting, and enhancing these networks, doing what is most meaningful for people, should be part of routine practice for palliative care professionals. Not doing so is not just an act of omission, it may actually cause harm.^23^

The remarkable facts to note in this section are as follows:

1. Terms such as community, civic, friendship, or neighbours fail to even rate a mention in this section.
2. The emphasis is firmly on assessment of patient and family needs but not the needs of their social networks to support the patient and their family – not workplaces, places of worship, schools, or social media.
3. Although there is a requirement to ‘discuss the impact of illness on interpersonal relationships’, there is no equally important exhortation to discuss the impact of interpersonal relationships on illness – and well-being.
4. Although trainees are expected to be familiar with a range of agencies that might support the disabled patient and their family, there is no similar injunction to be familiar with the informal and civic supports available to them.
5. Typical of ‘psychosocial’ texts most everywhere in palliative care, trainees are expected to ‘know about theoretical concepts of individuality’ but not those concerned with community.
6. Trainees are expected to use genograms to understand family relationships but they are not equally exhorted to use sociograms^24^ to understand community support and relationships – networks crucial to the support not only of patients but also the very families that form the basis of the required genograms.
7. There is a stipulation that trainees must learn to know when and how to use a family meeting but the same is not required for community meetings.
8. The trainee is expected to help ‘create environments that accommodate the needs of patients and families in the provision of palliative care’ but apparently not the civic environments that contextualize the family’s circumstances of support. The spaces referred to seem to be inpatient or home environments only. The ecology of care is fundamentally important in supporting patients, families, and friends at a time of stress and distress and includes place as well as relationships.^3,25^
9. There is no mention of one of the largest environments where the dying are increasingly found, aside from the usual hospitals, hospices, or home – nursing and care homes.
10. There is a stipulation that trainees are expected to ‘provide palliative care to the homeless and those in custody’, but no details are offered concerning the achievement of this goal.
11. The explicitly psychological sub-section of this psychosocial discussion emphasizes the need to describe impacts and responses to pain, intractable symptoms, uncertainty, loss, sadness, and depression, but there is no equal stipulation to discuss love, friendship, community support, meaning-making, well-being, or happiness and their impacts. And yet these uplifting experiences are as widely documented as negative experiences and are equally crucial in understanding individual adaptation and adjustment, coping, and health and well-being.
12. Regrettably, the heavy emphasis on negative themes continues throughout the section with trainees expected to ‘identify psychological responses as a source of additional problems and their role in obstructing the goals of care’. There is no equal stipulation that trainees should identify psychological responses as a potential source of solutions to problems or as helping to meet the goals of care.
13. The final section on grief and bereavement views these human experiences entirely negatively (there is no attempt to identify positive outcomes, assets, and attributes of the grief process) while the solution to sorrow is to ‘know about bereavement support and the organization of support services’. There is no mention of social assets – from friendship to workplaces and schools, from religious meaning and meetings, or to the healing role of community recognition and ritual. The bonds formed by caring networks often deepen and increase in number, and these last for years post bereavement and are an important part of how communities help to resolve grief.^26^

The Psychosocial section of the UK Palliative Medical Syllabus is bereft of public health and sociological concepts related to illness, caregiving, and grief and bereavement. From an academic public health point of view, and from the point of view of contemporary UK palliative care policy, this palliative medicine syllabus fails to deliver important current content and insight for its trainees.

## Concluding Reflections

The curriculum for UK Palliative Medicine will inevitably undergo revision – as all training documents do. When that time and process comes around again, we suggest that the public health approach to palliative care is embedded in the new curriculum. First, a wider circle of consultation will help avoid the omission and errors noted here. Medical colleagues from public health and geriatric medicine are crucial for a more informed public health framework and a more balanced understanding of both the epidemiology of ageing and dying as well as caregiving and bereavement. The omission of discussion and learning objectives related specifically to aged care is deeply unfortunate for a palliative care training document. Palliative care is not solely terminal cancer care. The clinical and policy demands for a broader involvement in ageing-related disease such as neurological disorders, organ failure, dementia, and even frailty have been debated and argued for over a decade now. The exponential rise in demographic ageing trends makes this attention even more urgent. Collaboration with gerontology can only strengthen the relevance and value of future palliative care learning and practice.

Second, it will be important to consult the established academic literature on health-promoting palliative care and to closely examine the existing palliative care policy documents for their stipulations and recommendations for social forms of support and care. Leadership and partnership are crucial new roles for palliative medicine in an age of the ‘new’ public health. Psychosocial approaches to care affirm the dominant direct service culture of palliative medicine and fail to direct attention to the crucial partnership challenges of working with community. These limits to psychosocial care are invitations to a greater understanding of health promotion and community development.

Finally, public health insights must be applied understandings for palliative care and not solely abstract epidemiological information bereft of practice guidance. Exhortations for promoting health in the general population, or to understand the epidemiology of life-limiting illness alone, are too easily interpreted as a form of death denial or death avoidance. There is irony here. Palliative medicine was born from the need to openly discuss dying, death, and loss, to bring these topics out of the shadows of medical over-treatment and the stigma of treatment failure. However, the absence of symptoms, including psychological and spiritual distress, is not the same as improvement in well-being. Palliative care can and should have a balanced approach towards harm reduction through control of symptoms of all kinds, but this should be balanced with a promotion of health and well-being through active enhancement of community support. In these ways, a strong public health approach to palliative care can provide not only a deeper understanding of the co-morbidities associated with mortality but also the insights from a well-being model that can help us and our patients to transcend them.
